# Epicardial ablation of ventricular tachycardia in a patient with arrhythmogenic right ventricular dysplasia after failed conventional endocardial ablation: A case for remote navigation with functional image integration

**DOI:** 10.21542/gcsp.2016.39

**Published:** 2016-12-30

**Authors:** Sabine Ernst, Karine Roy, Eric Lim, Glyn Thomas

**Affiliations:** 1 Department of Cardiology, Royal Brompton Hospital, London, UK; 2 NIHR Cardiovascular Biomedical Research Unit, Royal Brompton and National Heart and Lung Institute, Imperial College London, London, UK; 3 Department of Cardiology, University of Bristol, Bristol, UK

## Abstract

Arrhythmogenic right ventricular dysplasia (ARVD) is an inheritable heart muscle disease that predominantly affects the right ventricle (RV) and predisposes to ventricular arrhythmias and sudden cardiac death (SCD)^1^. The natural history is predominantly related to ventricular electric instability which may lead to arrhythmic SCD, mostly in young people and athletes^2,3^, but may progress with significant RV muscle disease and left-ventricular (LV) involvement and can result in right or biventricular heart failure^4^.

We report on a 54-year-old male with ARVD who underwent an epicardial ventricular tachycardia (VT) ablation using remote magnetic navigation (RMN) after functional imaging from a nuclear perfusion study was fused with a 3D segmentation from computed tomography (CT) imaging.

## Methods

**Figure 1.**
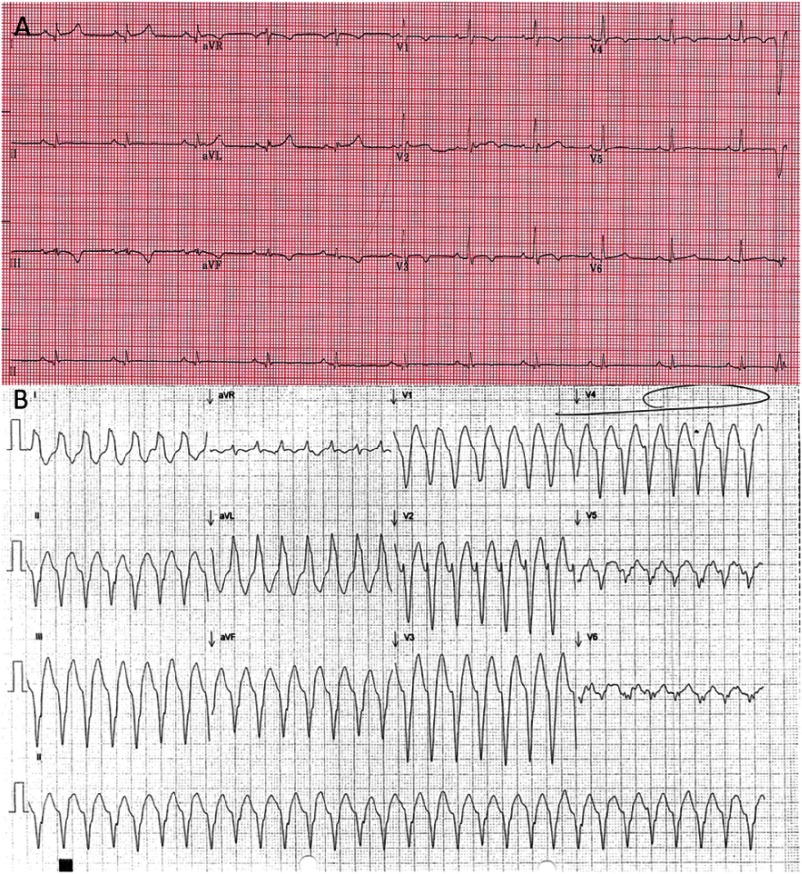
A) Sinus rhythm 12 lead ECG, B) 12 lead ECG during presentation to Accident and Emergency in ventricular tachycardia.

A 54-year-old male patient was diagnosed in 2011 with ARVD by following the standard criteria for diagnosis including late gadolinium enhancement in cardiac magnetic resonance imaging (CMR)^1^. Fig. 1 depicts the resting ECG in sinus rhythm (SR) (A) and during VT (B). Due to recurrent ventricular tachycardia he underwent an endocardial catheter ablation procedure and also had a dual chamber implantable cardioverter defibrillator (ICD) (Energen, Boston Scientific/Guidant) implanted. Unfortunately, he suffered from a number of further VT episodes, which resulted in shocks delivered after anti-tachycardia pacing accelerated the VT. A further two endocardial ablations were performed in February and August of 2015, but failed to control the VT sufficiently on ongoing Sotalol medication. Therefore an epicardial ablation procedure was electively planned including functional pre-procedural imaging and remote magnetic navigation.

### Pre-procedural imaging and image processing

**Figure 2.**
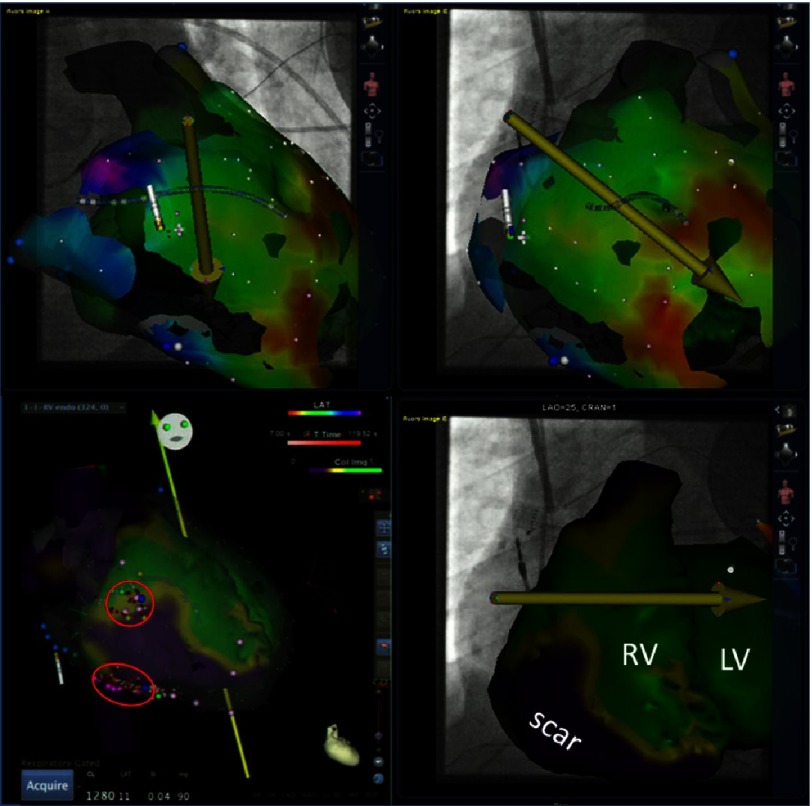
Top panels: Depiction of the epicardial mapping process using remote magnetic navigation. The catheter is moved in the epicardial space by moving the yellow vector in various directions and thereby changing the direction of the catheter tip. A mechanical drive allows advancement and retraction of the catheter and thereby free accessibility of the entire epicardial space. Bottom panels show the image integration of the fused images from nuclear (perfusion) and CT 3D segmentation. Green colour depicts normal perfusion, while purple depicts perfusion scar and yellow the border zone. Please note the normal LV perfusion (LV –EF is 66%), but the large perfusion scar of the RV. Purple tags mark late ventricular potentials.

**Figure 3.**
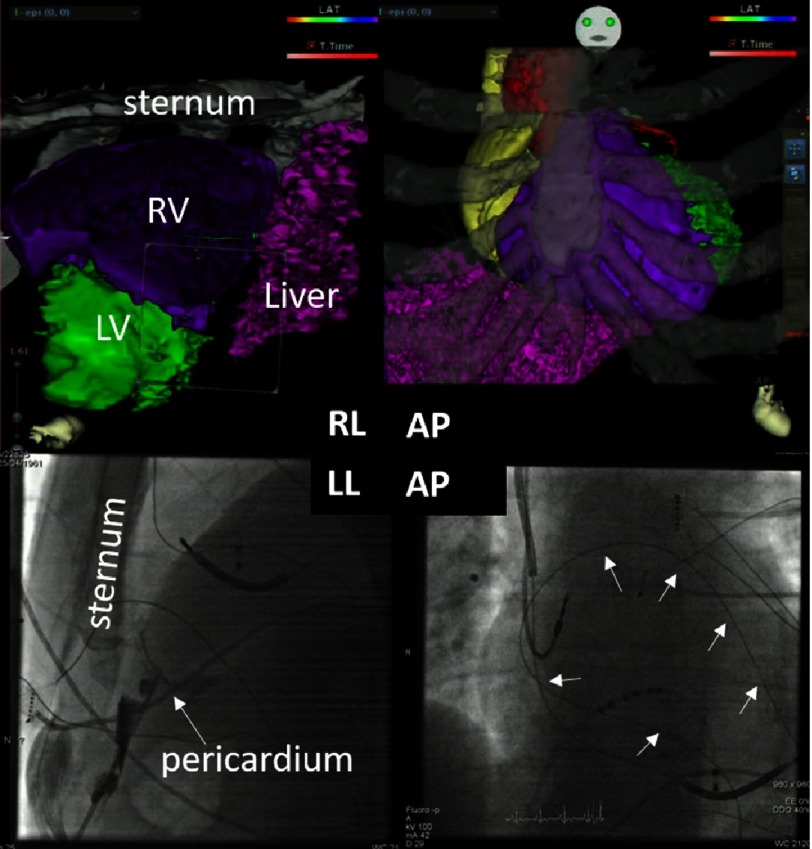
Top panels: 3D reconstruction of the cardiac anatomy in relation to the bony structures in anteroposterior (AP) and right lateral (RL) projection. The lower panels show the epicardial puncture using a Touchy needle and contrast injection (arrow), with subsequent wire insertion that wraps around the heart (small arrows) to exclude in-adverted access into the right ventricle.

Perfusion imaging was performed after resting injection of 480 MBq tetrofosmin on a dedicated cardiac Cadmium-Zinc-Tellur scanner (D-SPECT, Spectrum Dynamics, Israel)^5^ and fused with 3D segmentation from conventional contrast angiography CT (Fig. 2, bottom panels). In addition to the functional images, conventional “non-functional” 3D segmentation of the CT was performed with special reconstruction of the ribs and sternum to guide the epicardial puncture (Fig. 3). Both sets of images were used during the ablation procedure by integration with the electroanatomical mapping information (POLARIS software, Biosense Webster, Brussels, Belgium).

### Electrophysiologic study

After obtaining written informed consent, the procedure was performed under deep-assisted sedation in the presence of an experienced cardiac anaesthetist with continuous invasive arterial blood pressure monitoring. An octapolar diagnostic catheter (His/RV, custom-made, IBI) was positioned in the RV, serving as the timing and pacing reference via the right femoral vein (8 Fr sheath). The magnetically-equipped ablation catheter (Navistar ThermoCool RMT, Biosense Webster, Brussels, Belgium) was introduced either via the epicardially positioned sheath (Fig. 2) or via the femoral vein.

Intracardiac electrograms were recorded on a recording system (Axiom Sensis, Siemens, Erlangen, Germany) and all electrogram and mapping information was displayed on the Odyssey platform (Sterotaxis Inc., St. Louis, US). The magnetic navigation system (Niobe II, Sterotaxis Inc., St. Louis, US) was used in conjunction with the cardio drive system. A detailed description of this system has been published previously^6–8^.

Since the patient presented in SR at the beginning of the study, programmed stimulation was carried out in order to induce VT, which was hemodynamically not tolerated and required termination by overdrive pacing. Sequential electroanatomical mapping (CARTO 3 RMT, Biosense Webster) was performed during SR to acquire a substrate map and identify late potentials. Subsequently, pace-mapping was performed (using PASO, Biosense Webster, Israel) to identify exit sites for the various VTs (total of two sustained VTs with 270 ms and 240 ms cycle length, respectively). Multiple lesions at sites of best pacemap or at sites with late potentials (purple tags, Fig. 3) were deployed in the epicardial space (red circles), as well as from the endocardial side using irrigated tip radiofrequency current (45 Watts, 30 ml/sec flow, max 120 sec)^9,10^.

Final endpoint was the complete non-inducibility of any VT with up to 3 extras pacing from 2 different endocardial sites after the patient was completely awake. Hydrocortisone (10ml) was injected via the epicardial sheath, which was removed on the following day without evidence of epicardial effusion^11^. To reduce the risk of pericardial inflammation, oral Colchizine was recommended for 10 days^12^ and as well as continuation of the previously ineffective antiarrhythmic medication. The overall procedure duration amounted to 518 min with 5 min 2 sec of fluoroscopy time (623.7 Gycm2).

During a follow-up of more than 4 months, the patient has not experienced any further palpitations and no VT has been documented on his device via remote monitoring.

## Discussion

Patients with ARVD presenting with recurrent VT after endocardial ablation have a Class I indication for an epicardial ablation attempt in an experienced ablation centre^1,13,14^. Access to the pericardial space is nowadays a routine procedure and allows mapping of the epicardium, which presents in this patient cohort with significant substrate for re-entry tachycardia. Accessibility and mapping of the epicardial space is easily achieved using remote magnetic navigation. This has been demonstrated by several groups in various patient cohorts including ischemic and non-ischemic ventricular tachycardia^8,15,16^. The great advantage is that the operator is not exposed to scattered radiation and can manoeuvre the catheter without the risk of trauma around the ventricles (Fig. 2).

Further advantages can be taken by real time depiction of the ablation catheter on the fluoroscopic reference images. On these reference images, also the functional image fusion of nuclear perfusion imaging and computed tomography are displayed. Especially in patients with already implanted ICDs (even when MRI compatible) artefacts can make assessment of scar tissue by LGE particularly difficult^17^. In patients with ARVD, the expected scar/fibrosis area is very close to the RV coil such that fusion of the perfusion information from nuclear imaging superimposed on 3D segmented CT information offers an alternative. Of interest is that the borderzone of the depicted scar correlated very well with the 2 identified exit sites (ref. Fig. 3). This is in agreement to a recent publication from a different group, demonstrating good correlation to functional imaging from nuclear studies^18^. Further studies comparing the quality of information from functional nuclear+ CT versus LGE-MRI will be needed to quantify the additional advantage over the conventionally available “geometry-only” image merge techniques.

## Conclusion

Remote magnetic navigation allowed successful endo- and epicardial ablation of ventricular tachycardia in a patient with ARVD after failed conventional ablations. Functional image integration provided a substrate roadmap that allowed depiction of the epicardial scar and assisted in a very low radiation exposure.

## Funding support

This project was supported by the NIHR Cardiovascular Biomedical Research Unit of Royal Brompton and Harefield NHS Foundation Trust and Imperial College London.

## Disclosures

Sabine Ernst is a consultant to Biosense Webster and Stereotaxis, Inc.
